# Comparative Evaluation of Quercetin, Pioglitazone, Insulin, and Novel 5-Chromenyl–Methylene Thiazolidinedione Derivative on Nerve Function in Experimental Diabetic Peripheral Neuropathy

**DOI:** 10.3390/biomedicines14020418

**Published:** 2026-02-12

**Authors:** Adrian Haranguș, Irina Camelia Chiș, Simona Valeria Clichici, Camelia Alexandra Coadă, Remus Moldovan, Cristina Moldovan, Rareș Dumitru Ciocoi-Pop, Alina Toader, Laura Lele, Teodora Mocan

**Affiliations:** 1 Division of Physiology, Department of Morpho-Functional Sciences, “Iuliu Hațieganu” University of Medicine and Pharmacy, 1-3 Clinicilor Street, 400006 Cluj-Napoca, Romania; harangus_adrian@elearn.umfcluj.ro (A.H.); sclichici@umfcluj.ro (S.V.C.); coada_camelia_alexandra@elearn.umfcluj.ro (C.A.C.); toader.alina@umfcluj.ro (A.T.); teodoramocan@umfcluj.ro (T.M.); 2 Department of Pharmaceutical Chemistry, “Iuliu Hațieganu” University of Medicine and Pharmacy, 41 Victor Babeș Street, 400012 Cluj-Napoca, Romania; 3 Department of Physiotherapy and Theoretical Disciplines, Faculty of Physical Education and Sport, Babes-Bolyai University, 7 Pandurilor Street, 400376 Cluj-Napoca, Romania; rares.ciocoipop@ubbcluj.ro; 4 Department of Medical Disciplines, Faculty of Medicine and Pharmacy, University of Oradea, 101 Decembrie Street, Bihor County, 410073 Oradea, Romania; dr.laura.lele@gmail.com; 5 Department of Nanomedicine, Regional Institute of Gastroenterology and Hepatology, 5 Constanta Street, 400158 Cluj-Napoca, Romania

**Keywords:** animal model, diabetes, sciatic nerves, quercetin, pioglitazone, thiazolidine-2,4-dione, nerve conduction

## Abstract

**Background:** A debilitating complication of diabetes is diabetic peripheral neuropathy (DPN), for which effective therapy remains limited. In this research, we evaluated the effects of quercetin, pioglitazone, insulin, and a novel thiazolidine-2,4-dione derivative (TZDd) on the nerve functions in a streptozotocin (STZ)-induced rat model of DPN. **Methods:** In the experimental groups, STZ (60 mg/kg) was administered to Wistar rats to induce type 1 diabetic neuropathy, and the control and experimental DPN groups were treated with quercetin, pioglitazone, insulin, or TZDd for 5 weeks. The sensory and motor symptoms of DPN were evaluated via behavioral tests, nerve conduction velocity measurements, and electrophysiological assessment, and the synthesized TZDd was evaluated in silico for its pharmacokinetic, toxicological, and drug-likeness properties. **Results:** The diabetic rats developed DPN after 2 weeks of STZ administration, as evidenced by the significant reduction in the sensory and motor nerve conduction velocities (SNCVs and MNCVs) and increased mechanical hyperalgesia; on the other hand, quercetin, pioglitazone, insulin, and TZDd administration ameliorated the nerve functions of the DPN rats. In the in silico predictions, the novel TZDd exhibited no toxicity risks and demonstrated drug-like properties. **Conclusions:** Quercetin, pioglitazone, insulin, and TZDd showed neuroprotective effects that enhanced functional recovery in experimental DPN. These findings highlight that TZDd may represent a valuable compound with neuroprotective effects that could be used in DPN therapy and management.

## 1. Introduction

Diabetes mellitus (DM) is a complex endocrine disorder associated with severe vascular complications, including cardiovascular disease, nephropathy, retinopathy, and neuropathy [1,2,3]. In addition to hyperglycemia, factors such as insulin resistance, dyslipidemia, hypertension, thrombosis, systemic inflammation, oxidative stress, and abnormal protein kinase C activation contribute to DM complications [2,3]. Nearly half of DM patients develop diabetic peripheral neuropathy (DPN), a major neurodegenerative complication characterized by early sensory axon damage followed by motor fiber involvement [4]. DPN causes severe neuropathic pain, reduced quality of life, and an increased risk of chronic foot ulcers and lower-limb amputations, and it remains difficult to treat once established [5]. The multifactorial pathogenesis of DPN involves hyperglycemia, dyslipidemia, inflammation, nitro-oxidative stress, polyol pathway activation, mitochondrial dysfunction, toxic metabolite accumulation, and microvascular impairment leading to ischemia and axonal damage [1,2,3,4,5,6].

Several pathogenic mechanisms of DPN can be therapeutically targeted. Quercetin, a plant-derived flavonoid, exhibits hypoglycemic, anti-inflammatory, antioxidant, antiapoptotic, and neuroprotective effects, with multiple benefits in diseases such as cancer, cardiovascular disease, diabetes, and inflammation [7,8,9,10,11,12,13,14,15].

**Figure 1:**
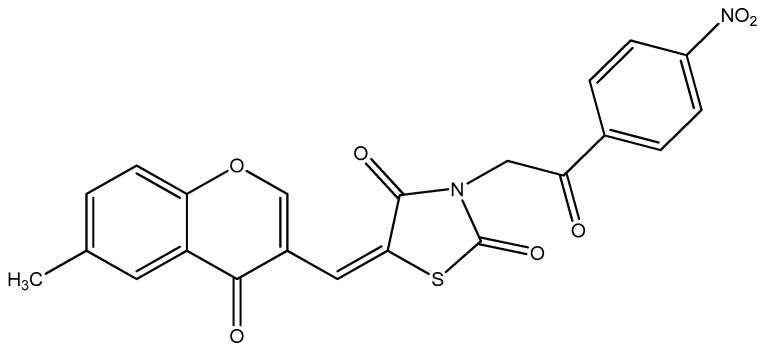
The chemical structure of the novel chromenyl-methylene-thiazolidine-2,4-dione derivative.

Thiazolidinediones, such as pioglitazone, improve insulin sensitivity and lipid metabolism, attenuate neuropathic pain behaviors, and modulate gene transcription involved in glucose and lipid homeostasis across multiple tissues [16,17,18,19,20,21,22,23,24]. Mechanistically, these effects are mediated primarily through the activation of peroxisome proliferator-activated receptor gamma (PPAR-γ), which plays a central role in regulating insulin sensitivity and lipid metabolism. However, their clinical use is limited by adverse effects, which has prompted the development of novel PPAR-γ agonists with improved efficacy and safety profiles [25,26]. Given the medicinal relevance of thiazolidine-2,4-dione derivatives (TZDds) and our experience in their development, in this study, we investigated the effects of a novel TZDd (Figure 1) [27,28,29] on the nerve function in rats with experimental DPN. The TZDd effects were compared with those of quercetin, pioglitazone, and insulin, alongside the in silico evaluation of the pharmacokinetic, toxicological, and drug-like properties and predicted PPAR-γ binding capacity.

A comparison of the effects of quercetin, pioglitazone, insulin, and a novel 5-chromenyl–methylene thiazolidinedione derivative in rats with experimental diabetes is scientifically justified because each agent represents a distinct therapeutic strategy in DPN. In this comparative design, the relative contributions of glycemic control, insulin sensitization, anti-inflammatory/antioxidant mechanisms, and structural innovation to nerve function improvement in experimental DPN are dissected. Moreover, this study positions the novel TZDd within the therapeutic landscape, investigating whether it offers advantages over the existing treatments or complementary effects.

## 2. Materials and Methods

### 2.1. Animal Care and Ethical Approval

One hundred male Wistar rats (three months old; body weights: 280–380 g) were acquired from the Biobase of “Iuliu Hatieganu” University of Medicine and Pharmacy, Cluj-Napoca, Romania. The rats were housed at the Animal House Laboratory at the Physiology Department under controlled laboratory conditions (12 h dark/light cycle; 21–23 °C; 50–60% humidity) with a standard rat pellet diet and water ad libitum. The animals were allowed one week of acclimatization prior to the start of the experiment.

The investigation was performed according to the International Animal Research Guidelines with the approval of the Ethical Committee on Animal Welfare (No. 413/20.08.2024) of the A.N.S.V.S.A. (National Sanitary Veterinary and Food Safety Authority), following the guidelines in the Use of Animals in Toxicology.

### 2.2. Experimental Design of Streptozotocin-Induced Diabetic Peripheral Neuropathy Rats

Animal models are the most reliable tools for evaluating pharmacological therapies prior to human clinical trials and remain central to preclinical drug development, a lengthy and costly process. DPN can be chemically induced using several experimental models, with STZ being the most common agent [30,31,32]. STZ is a nitrosourea derivative produced by *Streptomyces achromogenes* that selectively destroys pancreatic β-cells through DNA alkylation and macromolecular damage, resulting in hypoinsulinemia and hyperglycemia [31,32].

We induced type 1 diabetes mellitus (T1DM) in rats via the intraperitoneal (i.p.) injection of STZ (60 mg/kg body weight) (Sigma-Aldrich Chemical Company Inc.; St. Louis, MO, USA) dissolved in 0.1 mol/L sodium citrate buffer (pH: 4.5) [9,12,30,31,32]. The rats were treated intraperitoneally with 1 mL of 10% glucose solution thirty minutes after the STZ administration to prevent initial drug-induced hypoglycemic mortality [33], and the rats from the control groups received an equal-volume i.p. injection of citrate sodium buffer. Diabetes was confirmed 96 h after the STZ administration by measuring the fasting blood glucose (FBG) concentration. Rats with FBG levels higher than 250 mg/dL (13.89 mmol/L) were included in this study as diabetic rats.

Fasting blood glucose (FBG) analysis was performed via the glucose–oxidase method using a single drop of capillary blood sampled from the tail vein using an ACCU-CHEK Glucometer (Roche Diagnostics GmbH, Mannheim, Germany).

Neuropathy was confirmed two weeks after STZ injection based on mechanical hyperalgesia and reduced sensory and motor nerve conduction velocities in the hind paws of the animals, as previously reported by Malcangio et al. and Zhu et al. [30,34].

### 2.3. Animal Groups and Treatments

After developing neuropathy, the rats were divided into ten groups as follows:

Control groups:

Group C: control rats treated with carboxymethylcellulose (CMC);

Group Q: control rats treated with quercetin;

Group P: control rats treated with pioglitazone;

Group I: control rats treated with insulin;

Group T: control rats treated with TZDd;

Experimental DPN groups:

Group DC: DPN rats treated with CMC;

Group DQ: DPN rats treated with quercetin;

Group DP: DPN rats treated with pioglitazone;

Group DI: DPN rats treated with insulin;

Group DT: DPN rats treated with TZDd.

The group sizes were determined based on previously published MNCV measurements [35] comparing DPN rats and pioglitazone-treated animals, which show a moderate effect size of approximately 0.65. With a power of 0.8 and an adjusted significance level of 0.05, the calculated sample size was 9.1 animals per group, which was rounded up to 10 animals for each experimental arm.

The animals were randomly assigned to one of five treatment groups or their corresponding control groups using computer-generated randomization, which was stratified to ensure equal group sizes across all ten groups.

The investigators administering the treatments were aware of the group allocation due to the nature of the interventions; however, the outcome assessment and statistical analyses were conducted by investigators blinded to the group identities.

**Figure 2:**
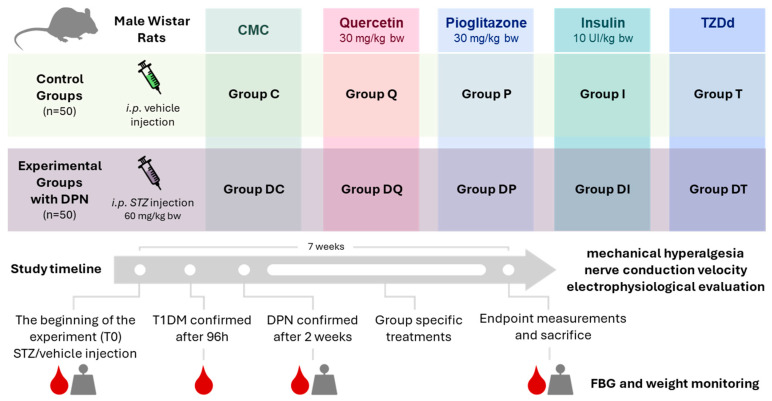
The experimental design of this study showing the treatment groups and measurement timepoints.

The CMC, quercetin, pioglitazone, insulin, and TZDd treatments were administered for 5 weeks in total. Two weeks after the STZ administration, DPN was confirmed in the rats, which were then administered CMC (0.6 mL/rat), quercetin (30 mg/kg body weight), and pioglitazone (30 mg/kg body weight) once daily via oral gavage, and TZDd was also administered via oral gavage in a dose equimolar to that of pioglitazone. Rats in Groups I and DI were subcutaneously injected with NovoRapid insulin at a dose of 10 UI/kg body weight/per day (Figure 2).

Then, the animals were included in a long-term study for 7 weeks. For the entire duration of the experiment, the following features were monitored in each animal: water consumption, mortality, and urine output daily, and food consumption and body weight weekly.

The FBG levels were measured in all animals at the beginning of the experiment (96 h), 2 weeks after the STZ administration, and at the end of the experiment (7 weeks after the STZ administration).

Following the treatment, all rats were anesthetized using an i.p. injection of 100/10 mg/kg ketamine/xylazine to prevent discomfort during the nerve conduction velocity measurements and electrophysiological assessments. At the end of the experiment (the 7th week of this study), after the behavioral pain testing, nerve conduction velocity measurements, and electrophysiological assessments, the animals were anesthetized with an i.p. sodium pentobarbital injection (60 mg/rat) and sacrificed via cervical dislocation.

### 2.4. Behavioral Tests: Measurement of Mechanical Hyperalgesia via Randall–Selitto Paw Withdrawal Test

The mechanical nociceptive threshold, an index of mechanical hyperalgesia, was measured in each animal to detect and quantify the neuropathic pain response in diabetic rats [36,37], and the thresholds were assessed symmetrically at both the hind paws and plantar surface using the Randall–Selitto Analgesy-Meter (Ugo Basile Analgesy-Meter, model 37215, Varese, Italy). The Randall–Selitto paw withdrawal test consisted of the application of an increasing mechanical force, for which the tip of the device was applied to both hind paws of the rat until a withdrawal response was elicited. For each hind paw, the procedure was repeated three times, twice with a 30 min interval between the two evaluations; the test was performed first on the left paw and then on the right, and the values were averaged. The nociceptive threshold is defined as the force (expressed in grams) at which the rat withdraws its paw [36,37]. Further details can be found in the Supplementary Materials.

### 2.5. Measurement of the Motor and Sensory Nerve Conduction Velocities

At week 7, the rats were anesthetized, the left hind limb shaved, and the core temperature maintained at 35–37 °C. Nerve conduction studies were performed using the MP150 Data Acquisition System (BIOPAC Systems, Goleta, CA, USA). Bipolar subcutaneous needle electrodes (0.5 mm × 20 mm) were used for stimulation and recording [38,39,40,41].

For both the MNCV and SNCV, the conduction velocity (m/s) was calculated as the distance between the two stimulation sites divided by the difference in the onset latencies. Further details regarding both the MNCV and SNCV are reported in the Supplementary Materials.

### 2.6. Electrophysiological Assessment

At week 7, the compound muscle action potentials (CMAPs) were recorded in anesthetized rats to assess the neuromuscular function [42], with the body temperatures of the rats maintained at 35–37 °C. A monopolar stimulation electrode was inserted 6–7 mm proximal to the sciatic nerve at the trochanter major, and an active recording electrode was positioned over the medial gastrocnemius muscle, with a reference electrode distally. Signals were amplified (bandpass: 1–10 Hz), digitized, and analyzed using the MP150 Biopac system, and the obtained CMAP values were averaged and compared across groups. A detailed presentation of the CMAP recording is reported in the Supplementary Materials.

### 2.7. In Silico Evaluation of TZDd Pharmacokinetic, Toxicological, and Drug-like Properties and PPAR-γ Binding Capacity

The lipophilicity, a key determinant of ADMET behavior, was evaluated using the n-octanol/water partition coefficient (LogP), which provides insight into the membrane permeability and plasma protein or receptor binding. The water solubility was assessed using the LogS, with LogS values near zero indicating good solubility and values below −4 indicating poor solubility. In addition, the interaction of the molecule with cytochrome P450 (CYP) isoenzymes was analyzed, as CYP inhibition is a major contributor to pharmacokinetic drug–drug interactions; therefore, predicting the likelihood and isoform specificity of CYP inhibition constitutes an essential step in early drug discovery profiling. All analyses were conducted using the SwissADME tool [43] and Osiris software [44], and the PPAR-γ binding capacities of TZDd and pioglitazone were quantified using the Enalos Cloud Platform [45].

### 2.8. Statistical Analysis

Statistical analysis was performed using GraphPad Prism, version 8.0 (San Diego, CA, USA). The data were reported as means ± standard deviations (SDs). One-way analysis of variance (ANOVA) was used to compare the differences between the groups, and two-way ANOVA was used for the repeated measurements, followed by Tukey’s multiple post hoc test, performed to compare the responses to CMC, quercetin, pioglitazone, insulin, and TZDs. Differences were considered significant if *p*-values were <0.05.

## 3. Results

### 3.1. Body Weights and Fasting Blood Glucose Levels in All Groups

The diabetic rats developed peripheral neuropathy after 2 weeks of STZ injection, as previously reported [30,34,46,47].

**Figure 3:**
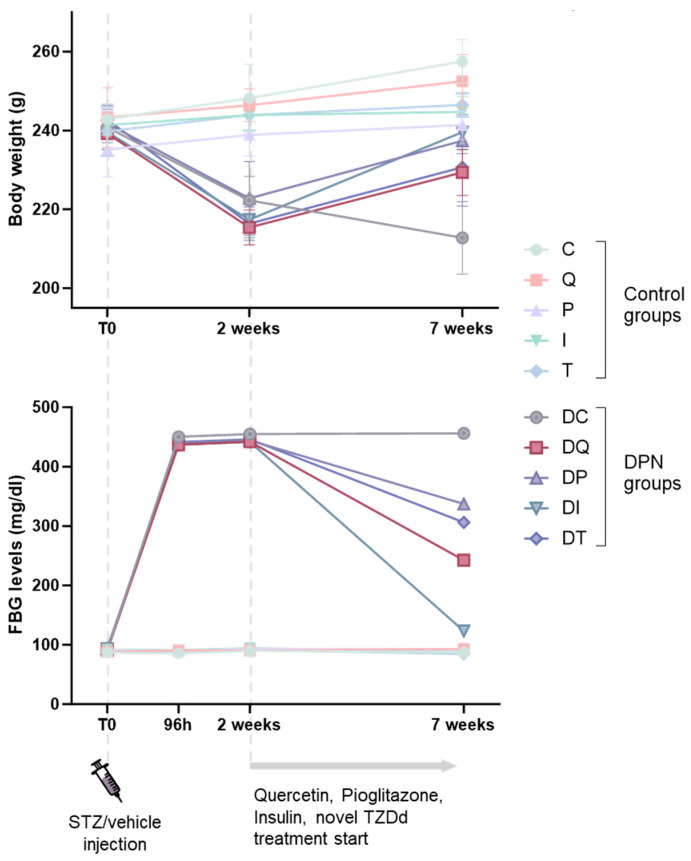
Effects of quercetin, pioglitazone, insulin, and thiazolidinedione derivative (TZDd) on body weights (BWs) and fasting blood glucose (FBG) levels of DPN-treated rats and corresponding controls. *n* = 10 animals per group. Error bars indicate SDs. Group C: control rats treated with carboxymethylcellulose (CMC); Group Q: control rats treated with quercetin; Group P: control rats treated with pioglitazone; Group I: control rats treated with insulin; Group T: control rats treated with TZDd; Group DC: DPN rats treated with CMC; Group DQ: DPN rats treated with quercetin; Group DP: DPN rats treated with pioglitazone; Group DI: DPN rats treated with insulin; Group DT: DPN rats treated with TZDd. SD: standard deviation.

The body weights and fasting blood glucose levels of all animals were measured at the beginning of the experiment, at specific timepoints after the STZ administration, and at the end of the experiment (i.e., 7 weeks after the STZ administration) (Figure 3; Supplementary Tables S1 and S2).

The Group C rats gained significant weight during the study period (*p* < 0.001), and the oral administration of quercetin, pioglitazone, or TZDd for 5 weeks did not impair normal weight gain in the animals. All the control group animals gained weight with respect to their first measurements (T0 vs. 7 weeks: C, *p* < 0.001; Q, *p* = 0.002; P, *p* = 0.050; T, *p* = 0.040), with the exception of those receiving insulin (I, *p* = 0.080) (Figure 3; Supplementary Tables S3 and S4).

At the beginning of the experiment, there were no significant differences in the body weights or FBG levels between the experimental DPN and control groups (Figure 3; Supplementary Tables S3 and S5). The body weights of the rats in the experimental DPN groups significantly decreased (*p* < 0.001) compared to those of the control groups 2 weeks after the STZ administration. However, the DQ and DT rats partially regained weight by the 7th experimental week (DQ vs. Q, *p* < 0.001; T vs. DT, *p* < 0.001), while the rats in the DP and DI groups completely recovered their initial weights, achieving similar values to those of their corresponding controls (DP vs. P, *p* = 0.860; DI vs. I, *p* = 0.590). On the contrary, the body weights of the rats in Group DC continued to decrease over time compared to those of the rats in Group C (*p* < 0.001) (Figure 3; Supplementary Tables S3 and S4).

The FBG levels of all animals were measured at the beginning of the experiment (96 h), 2 weeks after the STZ administration, and at the end of the experiment (i.e., 7 weeks after the STZ administration) (Figure 3; Supplementary Table S2).

The FBG levels were significantly increased (*p* < 0.001) in all the experimental DPN groups at 96 h and 2 weeks after the STZ administration compared with those of the Group C (Figure 3; Supplementary Tables S2, S5 and S6). After 5 weeks of the oral administration of quercetin, pioglitazone, insulin, or TZDd in DPN rats, the FBG levels decreased significantly when compared with those of Group DC (DQ vs. DC, *p* < 0.001; DP vs. DC, *p* = 0.004; DI vs. DC, *p* < 0.001; DT vs. DC, *p* = 0.007) (Supplementary Table S5).

### 3.2. Effects of Quercetin, Pioglitazone, Insulin, and Thiazolidinedione Derivative (TZDd) on Mechanical Hyperalgesia

**Figure 4:**
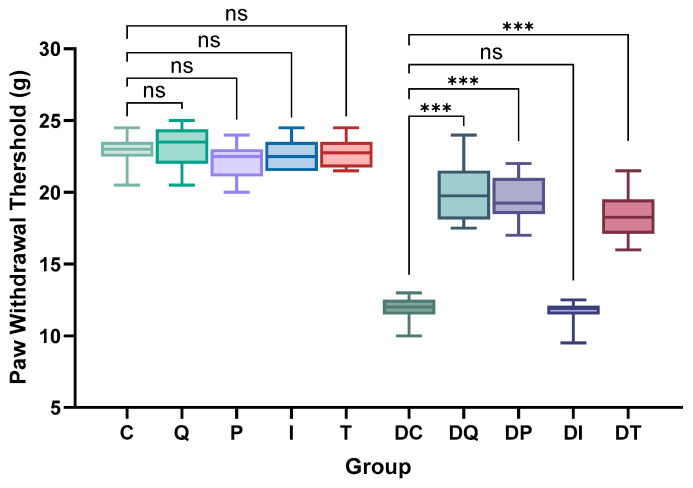
Effects of quercetin, pioglitazone, insulin, and thiazolidinedione derivative (TZDd) administration on mechanical nociceptive threshold (PWT) (g) in all groups measured at 7-week timepoint. *n* = 10 animals per group. Error bars indicate SDs. Group C: control rats treated with carboxymethylcellulose (CMC); Group Q: control rats treated with quercetin; Group P: control rats treated with pioglitazone; Group I: control rats treated with insulin; Group T: control rats treated with TZDd; Group DC: DPN rats treated with CMC; Group DQ: DPN rats treated with quercetin; Group DP: DPN rats treated with pioglitazone; Group DI: DPN rats treated with insulin; Group DT: DPN rats treated with TZDd. Results are representative of 10 rats per group. *** *p* < 0.001; ns: non-significant.

The mechanical nociceptive threshold was measured via the paw withdrawal threshold (PWT) test. Both the left and right paws were tested and showed similar results in each group (Supplementary Figure S1). Comparison between the groups was made on the merged left and right values. Administration of the drugs showed no effect on the paw withdrawal in the control groups, with similar values for all thresholds (*p* = ns) (Figure 4, Supplementary Table S7).

Group DC exhibited significant mechanical hyperalgesia 7 weeks after STZ administration compared with the controls (DC vs. C, *p* < 0.001). Quercetin, pioglitazone, and TZDd administration to STZ-treated rats significantly improved the mechanical nociceptive thresholds (DQ, DP, DT vs. DC, *p* < 0.001), although none managed to completely restore the normal sensitivity (DQ vs. Q; DP vs. P; DT vs. T, *p* < 0.001). Thus, while significant improvements were seen, the treatments did not ensure the full recovery of the measured parameters. Conversely, the insulin treatment had no effect on the paw withdrawal (DI vs. DC, *p* = 0.99) (Figure 4; Supplementary Figure S1, Supplementary Table S7).

### 3.3. Sensory Nerve Conduction Velocities in Internal Plantar Nerve and Motor Nerve Conduction Velocities in Sciatic Nerve

**Figure 5:**
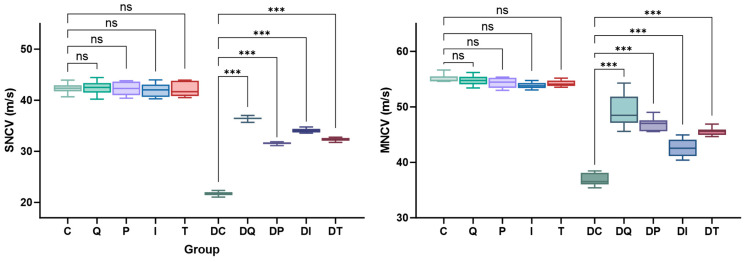
Effects of quercetin, pioglitazone, insulin, and thiazolidinedione derivative (TZDd) administration on sensory and motor nerve conduction velocities at 7-week timepoint. *n* = 10 animals per group. Error bars indicate SDs. Group C: control rats treated with carboxymethylcellulose (CMC); Group Q: control rats treated with quercetin; Group P: control rats treated with pioglitazone; Group I: control rats treated with insulin; Group T: control rats treated with TZDd; Group DC: DPN rats treated with CMC; Group DQ: DPN rats treated with quercetin; Group DP: DPN rats treated with pioglitazone; Group DI: DPN rats treated with insulin; Group DT: DPN rats treated with TZDd. *** *p* < 0.001; ns: non-significant.

The peripheral nerve function was assessed through SNCV and MNCV measurements, both of which significantly changed following the administered treatments. Namely, both the SNCV and MNCV were significantly decreased in Group DC with respect to the controls (Group C) (*p* < 0.001). All treatments improved the conduction velocities (DQ, DP, DI, DT vs. DC, *p* < 0.001), with the most significant effects seen in rats receiving quercetin (Figure 5; Supplementary Table S8). However, these treatments failed to completely restore the normal peripheral nerve function in this experimental setting, with significant differences still evident when comparing the treatment groups to their corresponding controls (DQ vs. Q; DP vs. P; DI vs. I; DT vs. T, all *p* < 0.001).

### 3.4. Electrophysiological Analysis—Compound Muscle Action Potential Measurements

**Figure 6:**
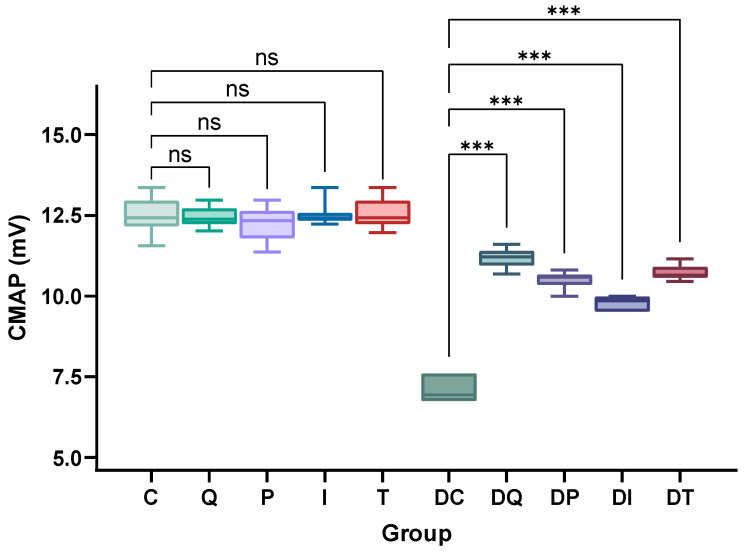
Effects of quercetin, pioglitazone, insulin, and thiazolidinedione derivative (TZDd) administration on average amplitude of evoked gastrocnemius muscle CMAPs. *n* = 10 animals per group. Error bars indicate SDs. Group C: control rats treated with carboxymethylcellulose (CMC); Group Q: control rats treated with quercetin; Group P: control rats treated with pioglitazone; Group I: control rats treated with insulin; Group T: control rats treated with TZDd; Group DC: DPN rats treated with CMC; Group DQ: DPN rats treated with quercetin; Group DP: DPN rats treated with pioglitazone; Group DI: DPN rats treated with insulin; Group DT: DPN rats treated with TZDd. *** *p* < 0.001; ns: non-significant.

The neuromuscular function was assessed using CMAP measurements. Group DC recorded significantly decreased CMAP values with respect to the controls (DC vs. C, *p* < 0.001) (Figure 6; Supplementary Table S9). All treatments significantly improved the neuromuscular function (DQ, DP, DI, DT vs. DC, *p* < 0.001), with the most potent effect seen in quercetin-treated animals. Nevertheless, this improvement was not sufficient to fully restore the neuromuscular function to the normal values obtained in the control groups (DQ vs. Q; DP vs. P; DI vs. I; DT vs. T, all *p* < 0.001) (Supplementary Table S9).

### 3.5. TZDd’s Pharmacokinetic, Toxicological, and Drug-like Properties and PPAR-γ Binding Capacity

According to the ADME prediction, TZDd seems to have low gastrointestinal absorption and does not cross the blood–brain barrier. The skin permeability was assessed via the LogKp, where less negative values indicate better absorption. The LogKp value was −6.48 cm/s, corresponding to poor skin penetration (Table 1). The predicted LogP of 2.72 and LogS of −4.95 indicate a lipophilic compound with good permeability and moderate solubility.

The synthetic TZDd is not a substrate for glycoprotein (P-gp) and therefore does not affect its permeability.

Our TZD derivative was predicted to be an inhibitor of three isoforms—CYP2C19, CYP2C9, and CYP3A4—with no inhibitory activity against two isoforms—CYP1A2 and CYP2D6.

Moreover, the pharmacokinetic properties of TZDd do not violate Lipinski’s rule of five, with a molecular weight < 500 Da, LogP < 5, number of hydrogen bond donors/acceptors < 5/10, and number of rotatable bonds < 10. Each of these parameters offers insights into a compound’s bioavailability, effectiveness, and safety profile.

Overall, these results highlight the promising ADME profile of TZDd and its predicted use safety, positioning it as a strong candidate for further optimization and development as a therapeutic agent.

In addition, the toxicity parameters were assessed using Osiris Property Explorer software (https://www.organic-chemistry.org/prog/peo, accessed on 18 November 2025), which showed no predicted toxicities, such as mutagenicity, carcinogenicity, or general toxicity (Table 1).

Considering the presence of thiazolidinedione, an important pharmacophore in the design of antidiabetic drugs that is also in the chemical structure of pioglitazone, we hypothesized that the TZDd effects of reducing hyperglycemia and ameliorating the negative changes in DPN are based on a similar mechanism of action to that of pioglitazone. The in silico evaluation of the PPAR-γ binding ability predicted a probability of 0.999 in binding receptors, while the binding score was 1 for pioglitazone. Therefore, the algorithm proposed the novel TZDd as a strong binder of PPAR-γ.

## 4. Discussion

In this research, we evaluated the neuroprotective effects of quercetin, pioglitazone, insulin, and a novel TZDd previously synthesized in our laboratory on a rat model of STZ-induced DPN. Quercetin, pioglitazone, and TZDd significantly attenuated the mechanical hyperalgesia, improved the motor and sensory nerve conduction velocities, and restored the compound muscle action potential amplitudes, whereas insulin exerted a limited effect on the neuropathic endpoints despite normalizing the blood glucose levels and partially improving the body weights.

These findings highlight the importance of mechanisms beyond glycemic control in the pathogenesis of DPN. While hyperglycemia is a critical initiating factor, others, such as oxidative stress, mitochondrial dysfunction, impaired Schwann cell metabolism, and microvascular ischemia, play central roles in the progression of neuropathy [1,2,3,4,5,6]. In line with this knowledge, we showed that agents with antioxidant, anti-inflammatory, or PPAR-γ agonist properties may provide superior neuroprotection compared with insulin monotherapy. Specifically, both quercetin and pioglitazone had significant neuroprotective and antinociceptive effects, resulting in the partial restoration of nerve conduction and muscle functionality. By contrast, insulin treatment alone failed to improve these parameters despite improving glycemia.

### 4.1. Body Weight and Glycemic Control

STZ administration induced a significant reduction in the body weights and a marked elevation in the FBG levels, consistent with previous reports describing STZ-induced β-cell destruction and insulin deficiency leading to catabolic weight loss and hyperglycemia [30,34,46,47]. As expected, insulin administration effectively reduced the FBG levels, while quercetin and pioglitazone also significantly ameliorated hyperglycemia after 5 weeks of treatment. Similar antihyperglycemic effects of quercetin have been reported in diabetic rodents, where quercetin enhanced the pancreatic antioxidant defenses and preserved the β-cell integrity [7,9,10]. Likewise, pioglitazone, a PPAR-γ agonist, is known to increase insulin sensitivity and improve glucose metabolism in STZ models [23].

Interestingly, the novel TZDd compound demonstrated a comparable glucose-lowering effect to that of pioglitazone, which, together with the strong PPAR-γ binding in silico prediction, suggests that it retains PPAR-γ activity and may confer similar metabolic benefits. Notably, all treatments except for insulin allowed normal weight gain in the controls and prevented progressive weight loss in DPN rats, indicating no impairment of normal metabolism or appetite. The partial recovery of the body weights in the DQ and DT groups can be induced via the metabolic stabilizing effects of these compounds. Previous findings [48] have also shown that quercetin and thiazolidinediones can reverse the weight loss associated with chronic diabetes.

### 4.2. Mechanical Hyperalgesia and Nociceptive Function

STZ-treated rats developed significant mechanical hyperalgesia, confirming successful DPN induction. Quercetin, pioglitazone, and TZDd administration markedly improved the paw withdrawal thresholds, indicating alleviation of the mechanical hypersensitivity. In contrast, the insulin treatment did not improve the mechanical sensitivity. These results align with prior studies reporting that quercetin attenuates hyperalgesia through antioxidant, anti-inflammatory, and neuroprotective mechanisms, including the suppression of NF-κB activation and the reduction in oxidative stress in peripheral nerves [49]. Similarly, pioglitazone has been shown to reduce neuropathic pain via the modulation of PPAR-γ-dependent pathways and the suppression of proinflammatory cytokines such as TNF-α and IL-6 [50]. The comparable efficacies of TZDd and pioglitazone suggest that although the TZDd synthesized by us may share common mechanisms related to PPAR-γ activation, it could possess additional antioxidant properties contributing to its neuroprotective effect.

### 4.3. Nerve Conduction and Electrophysiological Outcomes

The STZ model had significant SNCV and MNCV reductions, as well as decreased CMAPs, reflecting the axonal dysfunction and demyelination typical of DPN. Treatment with all the tested compounds significantly improved the nerve conduction parameters, although none fully restored the normal function. The greatest improvement was observed in the quercetin-treated rats, consistent with evidence that quercetin enhances myelin repair and improves mitochondrial function in peripheral nerves [51].

Insulin treatment improved the SNCVs, MNCVs, and neuromuscular functions, most likely due to its glycemic control and metabolic improvement effects. Pioglitazone and TZDd also improved the SNCVs and MNCVs, corroborating prior findings that PPAR-γ agonists promote neuronal survival, upregulate neurotrophic factors, and counteract oxidative damage in diabetic nerves [16,17,18,19,20,21,22,23,24]. The partial but significant recovery of the CMAP values in these groups indicates improved neuromuscular transmission, likely due to axonal integrity preservation and enhanced mitochondrial energy metabolism. The superior electrophysiological improvement observed with quercetin may be related to its dual role as a potent antioxidant and modulator of intracellular cascades, including AMPK and NF-kB signaling [52,53].

### 4.4. Integrated Interpretation and Mechanistic Insights

Collectively, these findings suggest that quercetin, pioglitazone, and TZDd ameliorate diabetic neuropathy via overlapping but distinct mechanisms. While pioglitazone and TZDd primarily act via the PPAR-γ-mediated enhancement of glucose and lipid metabolism, quercetin provides additional direct neuroprotective effects by scavenging free radicals, modulating inflammatory responses, and improving mitochondrial dynamics. Insulin improved nerve function most likely by reducing hyperglycemia and ensuring metabolic control. The novel 5-chromenyl–methylene TZDd hybrid, a disease-modifying candidate, combines the thiazolidinedione scaffold (linked to PPAR-γ-mediated metabolic effects) with a chromenyl moiety, potentially conferring additional antioxidant or neuroprotective properties. Comparing it to insulin, pioglitazone, and quercetin allows for an evaluation of the added benefit beyond glucose lowering, superiority or equivalence to an established TZD, and dual metabolic and neuroprotective actions.

### 4.5. TZDd’s Pharmacokinetic, Toxicological, and Drug-like Profile and PPAR-γ Binding Capacity

The combined ADMET analysis highlighted the novel TZDd as a promising drug candidate for further optimization and development as a therapeutic agent, with no toxicity risks, positioning it as a safe and effective drug candidate. The compound adhered to Lipinski’s rule of five, indicating a favorable pharmacokinetic profile, and it proved to be lipophilic, with excellent permeability but moderate water solubility (expressed by LogS), low gastrointestinal absorption, and low skin penetration, and it seems to not cross the blood–brain barrier, which may be an advantage for reducing possible adverse reactions in the central nervous system.

Moreover, TZDd was predicted not to be a substrate for the permeability glycoprotein (P-gp), which means that the molecule is less likely to be a cause of Pgp-related drug–drug interactions and therefore escapes the limitation of its concentration in plasma and, hence, its therapeutic effect. The compound was predicted as a strong binder of PPAR-γ.

### 4.6. Limitations and Future Perspectives

Although our study yielded encouraging results regarding the beneficial effects of quercetin, pioglitazone, and TZDd in improving the functional and electrophysiological parameters in a DPN rat model, some limitations are worth discussing. First, this study was initially designed to explore the clinical impacts of the selected treatments on the DPN degree; thus, we did not perform repeated measurements at multiple timepoints to show the evolution of the effects or their duration. Moreover, an extension beyond the 5-week time interval might be needed to allow for sufficient time for complete nerve recovery. Future studies should include histological analyses of nerve morphology and assessments of molecular markers of oxidative stress, inflammation, and mitochondrial function to help elucidate the mechanisms behind these positive findings. Additionally, dose–response and long-term administration studies would clarify whether quercetin and TZDd provide advantages over classical treatments, as well as establish the most efficient dosage for administration. The exclusive use of male animals also represents a limitation, as sex is a biological variable that may influence neuropathic outcomes and therapeutic efficacy. Combined treatments would also serve to explore whether quercetin and TZDd could exert synergistic effects with standard therapy, as well as to establish their utility in clinical settings. In sum, the potential of the tested treatments for DPN has yet to be explored.

## 5. Conclusions

Quercetin, pioglitazone, and the novel TZDd exerted significant protective effects in a STZ-induced model of DPN. The most evident improvements in the SNCVs and MNCVs, as well as in the mechanical nociceptive thresholds, were seen in the quercetin-treated animals, suggesting potent neuroprotective and antinociceptive actions of quercetin beyond its antihyperglycemic effects. Moreover, the beneficial outcomes observed in the TZDd-treated rats show favorable neuroprotective properties similar to those of pioglitazone. Overall, our findings highlight the therapeutic potential of quercetin, pioglitazone, insulin, and TZDd as adjunctive treatments for DPN.

## Figures and Tables

**Table 1:** ADME properties of synthesized thiazolidinedione derivative (TZDd) predicted via SwissADME.

Parameter Analyzed		Result
ADME Properties		
Physicochemical properties	Molecular weight (g/mol)	450
	Number of rotatable bonds	5
	Number of H-bond acceptors	7
	Number of H-bond donors	0
	TPSA (Å^2^)	155.78
Lipophilicity	Consensus LogP_*o*/*w*_	2.72
Water solubility	LogS (ESOL)	−4.95 (moderately soluble)
Pharmacokinetics	GI absorption	Low
	BBB permeant	No
	Log *K*_p_ (cm/s)	−6.48
	P-gp substrate	No
	CYP1A2 inhibitor	No
	CYP2C19 inhibitor	Yes
	CYP2C9 inhibitor	Yes
	CYP2D6 inhibitor	No
	CYP3A4 inhibitor	Yes
Toxicity Risk Predictions and Drug-Likeness Score		
Mutagenic		No
Tumorigenic		No
Irritant		No
Reproductive effect		No
Drug-likeness		Yes; 0 violations of Lipinski’s rules −6.97
Drug score		0.27

ADME: absorption, distribution, metabolism, and excretion; TPSA: topological polar surface area; GI: gastrointestinal; BBB: blood–brain barrier; P-gp: permeability glycoprotein.

## Data Availability

The original contributions presented in this study are included in the article/Supplementary Materials. Further inquiries can be directed to the corresponding authors.

## Supplementary Materials

The following supporting information can be downloaded at: https://www.mdpi.com/article/10.3390/biomedicines14020418/s1, Figure S1: Detailed presentation of measurements of left and right hind paws showing similar results between the left and right sides; Table S1: Effects of quercetin, pioglitazone, insulin, and thiazolidinedione derivative (TZDd) on body weight in DPN rats; Table S2: Effects of quercetin, pioglitazone, insulin, and thiazolidinedione derivative (TZDd) on fasting blood glucose (FBG) levels in DPN rats; Table S3: Comparison of the effects of quercetin, pioglitazone, insulin, and thiazolidinedione derivative (TZDd) on body weight in DPN rats; Table S4: Comparison of the effects of quercetin, pioglitazone, insulin, and thiazolidinedione derivative (TZDd) on animals body weight in DPN rats at different timepoints during the experiment; Table S5: Comparison of the effects of quercetin, pioglitazone, insulin, and thiazolidinedione derivative (TZDd) on blood glucose (FBG) in DPN rats; Table S6: Comparison of the effects of quercetin, pioglitazone, insulin, and thiazolidinedione derivative (TZDd) on blood glucose (FBG) in DPN rats at different timepoints during the experiment. Quercetin, Pioglitazone, Insulin, and TZDd were administered starting from week 2 after STZ injection; Table S7: Comparisons between groups with respect to their results in the paw withdrawal test (PWT) for mechanical nociceptive threshold determination. Measurements were done at week 7 after STZ/vehicle administration; Table S8: Comparisons between groups with respect to SNCV and MNCV measurements. Measurements were done at week 7 after STZ/vehicle administration; Table S9: Comparisons between groups with respect to CMAP measurements. Measurements were done at week 7 after STZ/vehicle administration.
