# Supplementary material for: Comparative Evaluation of Quercetin, Pioglitazone, Insulin, and Novel 5-Chromenyl–Methylene Thiazolidinedione Derivative on Nerve Function in Experimental Diabetic Peripheral Neuropathy
Source: Biomedicines. 2026 Feb 12;14(2):418. doi: 10.3390/biomedicines14020418 (PMC12937604; doi:10.3390/biomedicines14020418)
Supplement: Supplementary file 1 [file biomedicines-14-00418-s001.zip › biomedicines-4065796-supplementary.pdf]

Supplementary Methods

Drugs and Chemicals

5-((6-Methyl-4-oxo-4H-chromen-3-yl)methylene)-3-(2-(4-nitrophenyl)-2-oxoethyl)-thiazolidine-2,4-dione was previously synthesized in our laboratory and characterized physico-chemically [1].

STZ (cat. no. S0130-100 MG), pioglitazone hydrochloride (≥98% purity powder, cat. no. E6910-10MG), quercetin (3,3′,4′,5,7-pentahydroxyflavone dihydrate, >95% purity powder, cat. no. 337951-25G) were purchased from Sigma-Aldrich Chemical Company Inc. (St. Louis, USA). Treatments with quercetin, pioglitazone hydrochloride and TZDd were administered by oral gavage as 0.5% suspensions of the tested compounds, prepared in carboxymethylcellulose (CMC).

The NovoRapid Insulin (NovoRapid, INN-Insulin aspart) was purchased from Novo Nordisk A/S, Denmark.

Behavioral tests. Measurement of Mechanical Hyperalgesia by the Randall-Selitto Paw Withdrawal test

Mechanical nociceptive thresholds, were quantified in all animals in accordance to the instruction manual and previously published protocols [2,3], Analgesy-Meter Available online: https://ugobasile.com/products/categories/pain-and-inflammation/analgesy-meter (accessed on 31 January 2026). Rats undergoing this test were acclimated to handling starting 3 days before testing to reduce spontaneous movement during restraint. Before the test, each rat was placed in a chamber with a metal mesh floor and was habituated (5 minutes acclimation period) to manipulation; then it was placed into a soft cotton cloth and carefully immobilized with the same hand used to hold the tested paw. The experiments were carried out in a quiet room; all threshold tests were performed by the same person, at the same time of the day.

The test consisted of the application of an increasing mechanical force, in which the tip of the device was applied to the plantar surface of the rat hind paws until a withdrawal response resulted. The device exerted a force that increases steadily with 0.5 g/s, the maximum force of 350 g.

The Ugo Basil Analgesy-Meter has a pedal that is pressed by the experimenter, which determines the compression of the rat hind paw. The pressure at which a paw withdrawal occurred was recorded and this was taken as Paw Withdrawal Threshold (PWT). For each hind paw, the procedure was repeated three times, twice with a 30 min interval between the two evaluations, the test was performed first on the left and then on the right paw and the values were averaged. Successive stimuli were applied to alternating paws at 5 min intervals. All the experiments involving behavioral studies were performed by the experimenter who was unaware of treatments. Test was performed first on the left and then on the right paw and the values were averaged. The nociceptive threshold is defined as the force (expressed in grams) at which the rat withdraws its paw. The results represented the maximum force (expressed in grams) tolerated by the animals.

Measurement of the motor and sensory nerve conduction velocity

Motor nerve conduction velocities (MNCV) and sensory nerve conduction velocities (SNCV) in STZ-diabetic rats were measured with a MP150 Data Acquisition System (BIOPAC System Inc., USA) as previously described [4–7].

At the end of the experiment (7th week of study) all rats were anesthetized, and their left legs were shaved. During the measurements, the rats' body temperature was maintained between 35 and 37°C using an experimental hot plate.

For recording SNCV and MNCV bipolar needle electrodes (subcutaneous steel electrodes: 0.5 mm diameter, 20 mm length) were used. Nerve conduction velocity (recorded as meters per second) was calculated as the distance between the two stimulation points divided by the difference in the latency [4–7].

A small incision of approximately 2.5 cm was made at in the left sciatic notch and at the ankle to expose the left sciatic nerve of the rats and bipolar needle electrodes were placed at two different points.

For MNCV of the left sciatic motor nerve, a pair of stimulating electrodes were positioned at two different points on the left sciatic nerve. Proximal stimulating electrode (S1) was positioned in the left sciatic notch of the sciatic nerve, and the second stimulating electrode (S2) (distal electrode) was positioned in the ankle region of the left tibial nerve of the rats. The recording electrodes were placed in posterior plantar foot muscles. The stimulation intensity was 30 mA. The indifferent electrode was placed halfway between the stimulating and recording electrodes. The analysis period was 10 ms, the sensitivity 5 mV per division, and the bandpass was 2-2000 Hz. The distance (D) between stimulating and recording electrodes was measured. The difference between latencies (Δ latencies) recorded from proximal and distal stimulation sites was used to calculate the MNCV by the equation: MNCV (m/s) = D/ Δ latencies.

For SNCV of the left internal plantar nerve, a branch of the posterior tibial nerve that emerges to a superficial location at the external malleolus, the stimulating electrodes were placed in contact with the nerve on the inside of the tendon, 2 cm above the cuboid bone. The recording electrodes were placed at the level of the division of the digital nerve of the second space. The indifferent electrode was placed midway between the stimulating and recording electrodes. The frequency of stimulation was 1 Hz. The analysis period was 10 ms, the sensitivity 2 μV per division, and the bandpass 2-2000 Hz. The distance (D) between stimulating and recording electrodes was measured. The difference between latencies (Δ latencies) recorded from proximal and distal stimulation sites was used to calculate the SNCV by the equation: SNCV (m/s) = D/ Δ latencies

Electrophysiological assessment

The electrophysiological evaluation of the sciatic nerve was performed at the end of the study (7th week of the experiment) on all anaesthetized animals. Their left legs were shaved. During the measurements, the rats' body temperature was maintained between 35 and 37°C using an experimental hot plate.

Compound muscle action potentials (CMAP) for neuromuscular function assessment were recorded in the gastrocnemius muscle by using the EMG technique in the experimental and control groups as described previously [8].

The muscle activity was recorded with a monopolar needle electrode connected to an Ext-10C amplifier at the gastrocnemius muscle. Stimulus signals were generated, and signals were recorded using MP150 Biopac Student Lab Pro Version Data Acquisition System (BIOPAC System Inc., USA), which was also used for basic data analysis.

Under anesthesia, a monopolar needle stimulation electrode was placed 6–7 mm proximally to the sciatic nerve at the level of trochanter major. This is a reliable minimally invasive method for selectively eliciting CMAP. For recordings, an active electrode was positioned over the midpoint of the medial gastrocnemius muscle with the reference electrode at the lateral distal end of this muscle. CMAP peak-to-peak amplitude was recorded and averaged over a batch of 20 responses. The average amplitude in each group was compared with the control group. Recorded signals were amplified with band-pass filters between 1 Hz and 10 Hz and then analyzed by the BIOPAC Acknowledge Analysis Software.

ADME analysis

Lipophilicity is an important physicochemical descriptor, related to both pharmacodynamic and pharmacokinetic properties. It determines the absorption, metabolism, distribution, excretion and toxicity of drugs. It plays a significant role in the transport of molecules across membranes and the ability of compounds to bind to plasma proteins and/or receptors at the drug's action target. According to Lipinski's rule of five, lipophilicity is one of the most important factors determining the bioavailability of a drug. A value of logP > 5 is associated with undesirable characteristics, such as poor water solubility, tissue accumulation, fast metabolic turnover, or strong plasma protein binding. Water solubility was assessed by LogS, where values closer to 0 or positive suggest better solubility, while values below -4 indicate poor solubility.

Extremely important in drug discovery is the knowledge about interaction of molecules with cytochromes P450 (CYP). These isoenzymes have key roles in drug elimination through metabolic biotransformation. Their inhibition is one major cause of pharmacokinetics-related drug-drug interactions, leading to toxic or other unwanted adverse effects due to the lower clearance and accumulation of the drug or its metabolites. Contrarily, for some drugs, the association with an inhibitor is benefic, due to its accumulation which affords its dose reduction. It is therefore an important step in drug discovery the prediction of the predisposition with which the molecule will cause significant drug interactions through inhibition of CYPs, and also to determine which enzyme isoforms are affected.

Supplementary Results

Supplementary Table S1. Effects of quercetin, pioglitazone, insulin, and thiazolidinedione derivative (TZDd) on body weight in DPN rats.

	**Body weight**, mean±SD		
**Groups**	**The beginning of the experiment (T0) – before STZ/vehicle administration**	**2 weeks after STZ/vehicle administration***	**7 weeks after STZ/ vehicle administration***
Group C	242.9±3.9	248.2±8.6	257.5±5.6
Group Q	243.3±7.6	246.4±4.2	252.5±6.8
Group P	235.2±7	238.9±5.5	241.4±3.6
Group I	241.4±2.6	243.9±3.9	244.7±3.9
Group T	239.8±6.2	243.9±3.9	246.5±3

Group DC	240.8±5.5	222.3±6	212.8±9.2
Group DQ	239.2±5.6	215.4±4.5	229.4±5.9
Group DP	241.7±4.7	222.8±9.3	237.4±3.3
Group DI	239.6±4.5	217.4±4.6	239.6±4.5
Group DT	242.7±2.7	216.4±4.2	230.7±9.8

*The FBG levels (mg/dl) were monitored during the experiment: 96 h after STZ administration; 2 weeks after STZ administration and 7 weeks after STZ administration. Group C: control rats treated with carboxymethylcellulose (CMC); Group Q: control rats treated with quercetin; Group P: control rats treated with pioglitazone; Group I: control rats treated with insulin and Group T: control rats treated with TZDd; Group DC: DPN rats treated with CMC; Group DQ: DPN rats treated with quercetin; Group DP: DPN rats treated with pioglitazone; DI Group DI: DPN rats treated with insulin and Group DT: DPN rats treated with TZDd. *of note only DPN groups (DC, DQ, DP, DI, DT groups received STZ)*

Supplementary Table S2. Effects of quercetin, pioglitazone, insulin, and thiazolidinedione derivative (TZDd) on fasting blood glucose (FBG) levels in DPN rats.

		**FBG**, mean±SD		
**Groups**	**The beginning of the experiment (T0) – before STZ/vehicle administration**	**96 h after STZ/vehicle administration***	**2 weeks after STZ/vehicle administration***	**7 weeks after STZ/vehicle administration***
Group C	86.9±2.4	85.7±3.52	89.9±4.48	87.4±5.94
Group Q	90.3±1.5	90.6±3.02	94.7±3.56	93.4±2.17
Group P	91.3±2	89.6±3.16	93.5±3.02	91.4±1.77
Group I	92.5±4	91.2±4.6	95.3±2.6	87.4±3.8
Group T	91.7±1.8	88.3±3.09	91.5±4.00	84.8±3.52
Group DC	91.7±1.7	450.3±6.61	454.8±6.86	456.2±5.47
Group DQ	91.2±3.7	436.9±7.62	442±7.88	242.4±7.83
Group DP	94.4±1.5	440.9±8.17	444.3±5.90	337.3±4.54
Group DI	93.3±1.6	437.3±8.02	441.7±5.47	123.1±4.33
Group DT	90.4±1.6	441.3±5.98	445.9±6.75	306.1±7.69

*The FBG levels (mg/dl) were monitored at the beginning of the experiment – before treatment initiation (T0), during the experiment: 96 h after STZ administration; 2 weeks after STZ administration and 7 weeks after STZ administration. Group C: control rats treated with carboxymethylcellulose (CMC); Group Q: control rats treated with quercetin; Group P: control rats treated with pioglitazone; Group I: control rats treated with insulin and Group T: control rats treated with TZDd; Group DC: DPN rats treated with CMC; Group DQ: DPN rats treated with quercetin; Group DP: DPN rats treated with pioglitazone; DI Group DI: DPN rats treated with insulin and Group DT: DPN rats treated with TZDd. *of note only DPN groups (DC, DQ, DP, DI, DT groups received STZ)*

Supplementary Table S3. Comparison of the effects of quercetin, pioglitazone, insulin, and thiazolidinedione derivative (TZDd) on body weight in DPN rats.

	Body weight	
**Comparison**	**Mean Diff(95% CI)**	**adj. p-value**
**The beginning of the experiment (T0) – before STZ/vehicle administration**		
C vs. Q	-0.4(-5.39;4.59)	>0.99
C vs. P	7.7(2.71;12.7)	0.08
C vs. I	1.5(-3.49;6.49)	>0.99
C vs. T	3.1(-1.89;8.09)	0.97
C vs. DC	2.1(-2.89;7.09)	>0.99
C vs. DQ	3.7(-1.29;8.69)	0.91
C vs. DP	1.2(-3.79;6.19)	>0.99
C vs. DI	3.3(-1.69;8.29)	0.95
C vs. DT	0.2(-4.79;5.19)	>0.99
Q vs. P	8.1(3.11;13.1)	0.05
Q vs. I	1.9(-3.09;6.89)	>0.99
Q vs. T	3.5(-1.49;8.49)	0.93
Q vs. DC	2.5(-2.49;7.49)	>0.99
Q vs. DQ	4.1(-0.89;9.09)	0.84
Q vs. DP	1.6(-3.39;6.59)	>0.99
Q vs. DI	3.7(-1.29;8.69)	0.91
Q vs. DT	0.6(-4.39;5.59)	>0.99
P vs. I	-6.2(-11.2;-1.21)	0.30
P vs. T	-4.6(-9.59;0.39)	0.72
P vs. DC	-5.6(-10.6;-0.61)	0.45
P vs. DQ	-4(-8.99;0.991)	0.86
P vs. DP	-6.5(-11.5;-1.51)	0.24
P vs. DI	-4.4(-9.39;0.59)	0.77
P vs. DT	-7.5(-12.5;-2.51)	0.10
I vs. T	1.6(-3.39;6.59)	>0.99
I vs. DC	0.6(-4.39;5.59)	>0.99
I vs. DQ	2.2(-2.79;7.19)	>0.99
I vs. DP	-0.3(-5.29;4.69)	>0.99
I vs. DI	1.8(-3.19;6.79)	>0.99
I vs. DT	-1.3(-6.29;3.69)	>0.99
T vs. DC	-1(-5.99;3.99)	>0.99
T vs. DQ	0.6(-4.39;5.59)	>0.99
T vs. DP	-1.9(-6.89;3.09)	>0.99
T vs. DI	0.2(-4.79;5.19)	>0.99
T vs. DT	-2.9(-7.89;2.09)	0.98
DC vs. DQ	1.6(-3.39;6.59)	>0.99
DC vs. DP	-0.9(-5.89;4.09)	>0.99
DC vs. DI	1.2(-3.79;6.19)	>0.99

DC vs. DT	-1.9(-6.89;3.09)	>0.99
DQ vs. DP	-2.5(-7.49;2.49)	>0.99
DQ vs. DI	-0.4(-5.39;4.59)	>0.99
DQ vs. DT	-3.5(-8.49;1.49)	0.93
DP vs. DI	2.1(-2.89;7.09)	>0.99
DP vs. DT	-1(-5.99;3.99)	>0.99
DI vs. DT	-3.1(-8.09;1.89)	0.97
**2 weeks after STZ administration***		
C vs. Q	1.8(-3.19;6.79)	>0.99
C vs. P	9.3(4.31;14.3)	0.01
C vs. I	4.3(-0.691;9.29)	0.80
C vs. T	4.3(-0.691;9.29)	0.80
C vs. DC	25.9(20.9;30.9)	<0.001
C vs. DQ	32.8(27.8;37.8)	<0.001
C vs. DP	25.4(20.4;30.4)	<0.001
C vs. DI	30.8(25.8;35.8)	<0.001
C vs. DT	31.8(26.8;36.8)	<0.001
Q vs. P	7.5(2.51;12.5)	0.10
Q vs. I	2.5(-2.49;7.49)	>0.99
Q vs. T	2.5(-2.49;7.49)	>0.99
Q vs. DC	24.1(19.1;29.1)	<0.001
Q vs. DQ	31(26.0;36.0)	<0.001
Q vs. DP	23.6(18.6;28.6)	<0.001
Q vs. DI	29(24.0;34.0)	<0.001
Q vs. DT	30(25.0;35.0)	<0.001
P vs. I	-5(-9.99;-0.00915)	0.62
P vs. T	-5(-9.99;-0.00915)	0.62
P vs. DC	16.6(11.6;21.6)	<0.001
P vs. DQ	23.5(18.5;28.5)	<0.001
P vs. DP	16.1(11.1;21.1)	<0.001
P vs. DI	21.5(16.5;26.5)	<0.001
P vs. DT	22.5(17.5;27.5)	<0.001
I vs. T	0.01(-4.99;4.99)	>0.99
I vs. DC	21.6(16.6;26.6)	<0.001
I vs. DQ	28.5(23.5;33.5)	<0.001
I vs. DP	21.1(16.1;26.1)	<0.001
I vs. DI	26.5(21.5;31.5)	<0.001
I vs. DT	27.5(22.5;32.5)	<0.001
T vs. DC	21.6(16.6;26.6)	<0.001
T vs. DQ	28.5(23.5;33.5)	<0.001
T vs. DP	21.1(16.1;26.1)	<0.001
T vs. DI	26.5(21.5;31.5)	<0.001
T vs. DT	27.5(22.5;32.5)	<0.001

DC vs. DQ	6.9(1.91;11.9)	0.17
DC vs. DP	-0.5(-5.49;4.49)	>0.99
DC vs. DI	4.9(-0.09;9.89)	0.65
DC vs. DT	5.9(0.909;10.9)	0.37
DQ vs. DP	-7.4(-12.4;-2.41)	0.11
DQ vs. DI	-2(-6.99;2.99)	>0.99
DQ vs. DT	-1(-5.99;3.99)	>0.99
DP vs. DI	5.4(0.409;10.4)	0.51
DP vs. DT	6.4(1.41;11.4)	0.26
DI vs. DT	1(-3.99;5.99)	>0.99
**7 weeks after STZ administration***		
C vs. Q	5(0.00915;9.99)	0.62
C vs. P	16.1(11.1;21.1)	<0.001
C vs. I	12.8(7.81;17.8)	<0.001
C vs. T	11(6.01;16.0)	<0.001
C vs. DC	44.7(39.7;49.7)	<0.001
C vs. DQ	28.1(23.1;33.1)	<0.001
C vs. DP	20.1(15.1;25.1)	<0.001
C vs. DI	17.9(12.9;22.9)	<0.001
C vs. DT	26.8(21.8;31.8)	<0.001
Q vs. P	11.1(6.11;16.1)	<0.001
Q vs. I	7.8(2.81;12.8)	0.07
Q vs. T	6(1.01;11.0)	0.35
Q vs. DC	39.7(34.7;44.7)	<0.001
Q vs. DQ	23.1(18.1;28.1)	<0.001
Q vs. DP	15.1(10.1;20.1)	<0.001
Q vs. DI	12.9(7.91;17.9)	<0.001
Q vs. DT	21.8(16.8;26.8)	<0.001
P vs. I	-3.3(-8.29;1.69)	0.95
P vs. T	-5.1(-10.1;-0.109)	0.59
P vs. DC	28.6(23.6;33.6)	<0.001
P vs. DQ	12(7.01;17.0)	<0.001
P vs. DP	4(-0.991;8.99)	0.86
P vs. DI	1.8(-3.19;6.79)	>0.99
P vs. DT	10.7(5.71;15.7)	0.001
I vs. T	-1.8(-6.79;3.19)	>0.99
I vs. DC	31.9(26.9;36.9)	<0.001
I vs. DQ	15.3(10.3;20.3)	<0.001
I vs. DP	7.3(2.31;12.3)	0.12
I vs. DI	5.1(0.109;10.1)	0.59
I vs. DT	14(9.01;19.0)	<0.001
T vs. DC	33.7(28.7;38.7)	<0.001
T vs. DQ	17.1(12.1;22.1)	<0.001

T vs. DP	9.1(4.11;14.1)	0.01
T vs. DI	6.9(1.91;11.9)	0.17
T vs. DT	15.8(10.8;20.8)	<0.001
DC vs. DQ	-16.6(-21.6;-11.6)	<0.001
DC vs. DP	-24.6(-29.6;-19.6)	<0.001
DC vs. DI	-26.8(-31.8;-21.8)	<0.001
DC vs. DT	-17.9(-22.9;-12.9)	<0.001
DQ vs. DP	-8(-13.0;-3.01)	0.06
DQ vs. DI	-10.2(-15.2;-5.21)	0.003
DQ vs. DT	-1.3(-6.29;3.69)	>0.99
DP vs. DI	-2.2(-7.19;2.79)	>0.99
DP vs. DT	6.7(1.71;11.7)	0.20
DI vs. DT	8.9(3.91;13.9)	0.02

Supplementary Table S4. Comparison of the effects of quercetin, pioglitazone, insulin, and thiazolidinedione derivative (TZDd) on animals body weight in DPN rats at different timepoints during the experiment.

Group	Mean Diff(95% CI)	adj. p-value
**C**		
T0 vs. 2 weeks	-5.3(-11.4 to 0.846)	0.09
T0 vs. 7 weeks	-14.6(-19.0 to -10.2)	<0.001
2 weeks vs. 7 weeks	-9.3(-16.0 to -2.63)	0.009
**Q**		
T0 vs. 2 weeks	-3.1(-9.47 to 3.27)	0.4
T0 vs. 7 weeks	-9.2(-14.4 to -3.97)	0.002
2 weeks vs. 7 weeks	-6.1(-11.5 to -0.682)	0.03
**P**		
T0 vs. 2 weeks	-3.7(-6.70 to -0.697)	0.02
T0 vs. 7 weeks	-6.2(-12.5 to 0.109)	0.05
2 weeks vs. 7 weeks	-2.5(-7.99 to 2.99)	0.45
**I**		
T0 vs. 2 weeks	-2.5(-6.30 to 1.30)	0.21
T0 vs. 7 weeks	-3.3(-6.95 to 0.353)	0.08
2 weeks vs. 7 weeks	-0.8(-2.35 to 0.746)	0.36
**T**		
T0 vs. 2 weeks	-4.1(-10.8 to 2.64)	0.26
T0 vs. 7 weeks	-6.7(-13.1 to -0.251)	0.04
2 weeks vs. 7 weeks	-2.6(-5.80 to 0.602)	0.11
**DC**		
T0 vs. 2 weeks	18.5(15.5 to 21.5)	<0.001
T0 vs. 7 weeks	28(22.4 to 33.6)	<0.001
2 weeks vs. 7 weeks	9.5(6.24 to 12.8)	<0.001
**DQ**		
T0 vs. 2 weeks	23.8(19.4 to 28.2)	<0.001

T0 vs. 7 weeks	9.8(1.46 to 18.1)	0.02
2 weeks vs. 7 weeks	-14(-21.5 to -6.50)	0.001
**DP**		
T0 vs. 2 weeks	18.9(9.19 to 28.6)	0.001
T0 vs. 7 weeks	4.3(-1.67 to 10.3)	0.17
2 weeks vs. 7 weeks	-14.6(-22.0 to -7.19)	<0.001
**DI**		
T0 vs. 2 weeks	22.2(18.1 to 26.3)	<0.001
T0 vs. 7 weeks	0.1(-0.401 to 0.601)	0.85
2 weeks vs. 7 weeks	-22.2(-26.3 to -18.1)	<0.001
**DT**		
T0 vs. 2 weeks	26.3(23.2 to 29.4)	<0.001
T0 vs. 7 weeks	12(3.89 to 20.1)	0.006
2 weeks vs. 7 weeks	-14.3(-22.6 to -5.96)	0.003

Supplementary Table S5. Comparison of the effects of quercetin, pioglitazone, insulin, and thiazolidinedione derivative (TZDd) on blood glucose (FBG) in DPN rats.

	**Blood glucose**	
**Comparison**	**Mean Diff(95% CI)**	**adj. p-value**
**The beginning of the experiment (T0) – before STZ/vehicle administration**		
C vs. Q	-1.1(-7.95 to 5.75)	>0.99
C vs. P	-4.4(-11.2 to 2.45)	0.57
C vs. I	-5.6(-12.4 to 1.25)	0.22
C vs. T	-4.8(-11.6 to 2.05)	0.44
C vs. DC	-4.8(-11.6 to 2.05)	0.44
C vs. DQ	-4.3(-11.1 to 2.55)	0.6
C vs. DP	-7.5(-14.3 to -0.651)	0.02
C vs. DI	-6.4(-13.2 to 0.449)	0.09
C vs. DT	-3.5(-10.3 to 3.35)	0.83
Q vs. P	-3.3(-10.1 to 3.55)	0.88
Q vs. I	-4.5(-11.3 to 2.35)	0.53
Q vs. T	-3.7(-10.5 to 3.15)	0.78
Q vs. DC	-3.7(-10.5 to 3.15)	0.78
Q vs. DQ	-3.2(-10.0 to 3.65)	0.9
Q vs. DP	-6.4(-13.2 to 0.449)	0.09
Q vs. DI	-5.3(-12.1 to 1.55)	0.29
Q vs. DT	-2.4(-9.25 to 4.45)	0.98
P vs. I	-1.2(-8.05 to 5.65)	>0.99
P vs. T	-0.4(-7.25 to 6.45)	>0.99
P vs. DC	-0.4(-7.25 to 6.45)	>0.99

P vs. DQ	0.1(-6.75 to 6.95)	>0.99
P vs. DP	-3.1(-9.95 to 3.75)	0.91
P vs. DI	-2(-8.85 to 4.85)	>0.99
P vs. DT	0.9(-5.95 to 7.75)	>0.99
I vs. T	0.8(-6.05 to 7.65)	>0.99
I vs. DC	0.8(-6.05 to 7.65)	>0.99
I vs. DQ	1.3(-5.55 to 8.15)	>0.99
I vs. DP	-1.9(-8.75 to 4.95)	>0.99
I vs. DI	-0.8(-7.65 to 6.05)	>0.99
I vs. DT	2.1(-4.75 to 8.95)	>0.99
T vs. DC	0.01(-6.85 to 6.85)	>0.99
T vs. DQ	0.5(-6.35 to 7.35)	>0.99
T vs. DP	-2.7(-9.55 to 4.15)	0.96
T vs. DI	-1.6(-8.45 to 5.25)	>0.99
T vs. DT	1.3(-5.55 to 8.15)	>0.99
DC vs. DQ	0.5(-6.35 to 7.35)	>0.99
DC vs. DP	-2.7(-9.55 to 4.15)	0.96
DC vs. DI	-1.6(-8.45 to 5.25)	>0.99
DC vs. DT	1.3(-5.55 to 8.15)	>0.99
DQ vs. DP	-3.2(-10.0 to 3.65)	0.9
DQ vs. DI	-2.1(-8.95 to 4.75)	>0.99
DQ vs. DT	0.8(-6.05 to 7.65)	>0.99
DP vs. DI	1.1(-5.75 to 7.95)	>0.99
DP vs. DT	4(-2.85 to 10.8)	0.7
DI vs. DT	2.9(-3.95 to 9.75)	0.94
**96h after STZ/vehicle administration***		
C vs. Q	-4.9(-11.7 to 1.95)	0.41
C vs. P	-3.9(-10.7 to 2.95)	0.73
C vs. I	-5.5(-12.3 to 1.35)	0.24
C vs. T	-2.6(-9.45 to 4.25)	0.97
C vs. DC	-365(-371 to -358)	<0.001
C vs. DQ	-351(-358 to -344)	<0.001
C vs. DP	-355(-362 to -348)	<0.001
C vs. DI	-352(-358 to -345)	<0.001
C vs. DT	-356(-362 to -349)	<0.001
Q vs. P	1(-5.85 to 7.85)	>0.99
Q vs. I	-0.6(-7.45 to 6.25)	>0.99
Q vs. T	2.3(-4.55 to 9.15)	0.99
Q vs. DC	-360(-367 to -353)	<0.001
Q vs. DQ	-346(-353 to -339)	<0.001
Q vs. DP	-350(-357 to -343)	<0.001
Q vs. DI	-347(-354 to -340)	<0.001
Q vs. DT	-351(-358 to -344)	<0.001

P vs. I	-1.6(-8.45 to 5.25)	>0.99
P vs. T	1.3(-5.55 to 8.15)	>0.99
P vs. DC	-361(-368 to -354)	<0.001
P vs. DQ	-347(-354 to -340)	<0.001
P vs. DP	-351(-358 to -344)	<0.001
P vs. DI	-348(-355 to -341)	<0.001
P vs. DT	-352(-359 to -345)	<0.001
I vs. T	2.9(-3.95 to 9.75)	0.94
I vs. DC	-359(-366 to -352)	<0.001
I vs. DQ	-346(-353 to -339)	<0.001
I vs. DP	-350(-357 to -343)	<0.001
I vs. DI	-346(-353 to -339)	<0.001
I vs. DT	-350(-357 to -343)	<0.001
T vs. DC	-362(-369 to -355)	<0.001
T vs. DQ	-349(-355 to -342)	<0.001
T vs. DP	-353(-359 to -346)	<0.001
T vs. DI	-349(-356 to -342)	<0.001
T vs. DT	-353(-360 to -346)	<0.001
DC vs. DQ	13.4(6.55 to 20.2)	<0.001
DC vs. DP	9.4(2.55 to 16.2)	<0.001
DC vs. DI	13(6.15 to 19.8)	<0.001
DC vs. DT	9(2.15 to 15.8)	0.001
DQ vs. DP	-4(-10.8 to 2.85)	0.7
DQ vs. DI	-0.4(-7.25 to 6.45)	>0.99
DQ vs. DT	-4.4(-11.2 to 2.45)	0.57
DP vs. DI	3.6(-3.25 to 10.4)	0.81
DP vs. DT	-0.4(-7.25 to 6.45)	>0.99
DI vs. DT	-4(-10.8 to 2.85)	0.7
**2 weeks after STZ/vehicle administration***		
C vs. Q	-0.5(-7.35 to 6.35)	>0.99
C vs. P	-3.6(-10.4 to 3.25)	0.81
C vs. I	-5.4(-12.2 to 1.45)	0.27
C vs. T	-1.6(-8.45 to 5.25)	>0.99
C vs. DC	-365(-372 to -358)	<0.001
C vs. DQ	-352(-359 to -345)	<0.001
C vs. DP	-354(-361 to -348)	<0.001
C vs. DI	-352(-359 to -345)	<0.001
C vs. DT	-356(-363 to -349)	<0.001
Q vs. P	-3.1(-9.95 to 3.75)	0.91
Q vs. I	-4.9(-11.7 to 1.95)	0.41
Q vs. T	-1.1(-7.95 to 5.75)	>0.99
Q vs. DC	-364(-371 to -358)	<0.001
Q vs. DQ	-352(-358 to -345)	<0.001

Q vs. DP	-354(-361 to -347)	<0.001
Q vs. DI	-351(-358 to -344)	<0.001
Q vs. DT	-356(-362 to -349)	<0.001
P vs. I	-1.8(-8.65 to 5.05)	>0.99
P vs. T	2(-4.85 to 8.85)	>0.99
P vs. DC	-361(-368 to -354)	<0.001
P vs. DQ	-349(-355 to -342)	<0.001
P vs. DP	-351(-358 to -344)	<0.001
P vs. DI	-348(-355 to -341)	<0.001
P vs. DT	-352(-359 to -346)	<0.001
I vs. T	3.8(-3.05 to 10.6)	0.76
I vs. DC	-360(-366 to -353)	<0.001
I vs. DQ	-347(-354 to -340)	<0.001
I vs. DP	-349(-356 to -342)	<0.001
I vs. DI	-346(-353 to -340)	<0.001
I vs. DT	-351(-357 to -344)	<0.001
T vs. DC	-363(-370 to -356)	<0.001
T vs. DQ	-351(-357 to -344)	<0.001
T vs. DP	-353(-360 to -346)	<0.001
T vs. DI	-350(-357 to -343)	<0.001
T vs. DT	-354(-361 to -348)	<0.001
DC vs. DQ	12.8(5.95 to 19.6)	<0.001
DC vs. DP	10.5(3.65 to 17.3)	<0.001
DC vs. DI	13.1(6.25 to 19.9)	<0.001
DC vs. DT	8.9(2.05 to 15.7)	0.002
DQ vs. DP	-2.3(-9.15 to 4.55)	0.99
DQ vs. DI	0.3(-6.55 to 7.15)	>0.99
DQ vs. DT	-3.9(-10.7 to 2.95)	0.73
DP vs. DI	2.6(-4.25 to 9.45)	0.97
DP vs. DT	-1.6(-8.45 to 5.25)	>0.99
DI vs. DT	-4.2(-11.0 to 2.65)	0.63
**7 weeks after STZ/vehicle administration***		
C vs. Q	-6(-12.8 to 0.849)	0.14
C vs. P	-4(-10.8 to 2.85)	0.7
C vs. I	0(-6.85 to 6.85)	>0.99
C vs. T	2.6(-4.25 to 9.45)	0.97
C vs. DC	-369(-376 to -362)	<0.001
C vs. DQ	-155(-162 to -148)	<0.001
C vs. DP	-250(-257 to -243)	<0.001
C vs. DI	-35.7(-42.5 to -28.9)	<0.001
C vs. DT	-219(-226 to -212)	<0.001
Q vs. P	2(-4.85 to 8.85)	>0.99
Q vs. I	6(-0.849 to 12.8)	0.14

Q vs. T	8.6(1.75 to 15.4)	0.003
Q vs. DC	-363(-370 to -356)	<0.001
Q vs. DQ	-149(-156 to -142)	<0.001
Q vs. DP	-244(-251 to -237)	<0.001
Q vs. DI	-29.7(-36.5 to -22.9)	<0.001
Q vs. DT	-213(-220 to -206)	<0.001
P vs. I	4(-2.85 to 10.8)	0.7
P vs. T	6.6(-0.249 to 13.4)	0.07
P vs. DC	-365(-372 to -358)	<0.001
P vs. DQ	-151(-158 to -144)	<0.001
P vs. DP	-246(-253 to -239)	<0.001
P vs. DI	-31.7(-38.5 to -24.9)	<0.001
P vs. DT	-215(-222 to -208)	<0.001
I vs. T	2.6(-4.25 to 9.45)	0.97
I vs. DC	-369(-376 to -362)	<0.001
I vs. DQ	-155(-162 to -148)	<0.001
I vs. DP	-250(-257 to -243)	<0.001
I vs. DI	-35.7(-42.5 to -28.9)	<0.001
I vs. DT	-219(-226 to -212)	<0.001
T vs. DC	-371(-378 to -365)	<0.001
T vs. DQ	-158(-164 to -151)	<0.001
T vs. DP	-253(-259 to -246)	<0.001
T vs. DI	-38.3(-45.1 to -31.5)	<0.001
T vs. DT	-221(-228 to -214)	<0.001
DC vs. DQ	214(207 to 221)	<0.001
DC vs. DP	119(112 to 126)	<0.001
DC vs. DI	333(326 to 340)	<0.001
DC vs. DT	150(143 to 157)	<0.001
DQ vs. DP	-94.9(-102 to -88.1)	<0.001
DQ vs. DI	119(112 to 126)	<0.001
DQ vs. DT	-63.7(-70.5 to -56.9)	<0.001
DP vs. DI	214(207 to 221)	<0.001
DP vs. DT	31.2(24.4 to 38.0)	<0.001
DI vs. DT	-183(-190 to -176)	<0.001

Supplementary Table S6. Comparison of the effects of quercetin, pioglitazone, insulin, and thiazolidinedione derivative (TZDd) on blood glucose (FBG) in DPN rats at different timepoints during the experiment. Quercetin, Pioglitazone, Insulin, and TZDd were administered starting from week 2 after STZ injection.

Group	Mean Diff(95% CI)	adj. p-value
**C**		
T0 vs. 96h	1.2(-4.32 to 6.72)	0.9

T0 vs. 2 weeks	-3(-9.03 to 3.03)	0.45
T0 vs. 7 weeks	-0.5(-7.39 to 6.39)	>0.99
2 weeks vs. 7 weeks	2.5(-1.69 to 6.69)	0.31
**Q**		
T0 vs. 96h	-2.6(-7.21 to 2.01)	0.35
T0 vs. 2 weeks	-2.4(-5.42 to 0.62)	0.13
T0 vs. 7 weeks	-5.4(-8.73 to -2.07)	0.003
2 weeks vs. 7 weeks	-3(-7.61 to 1.61)	0.25
**P**		
T0 vs. 96h	1.7(-2.41 to 5.81)	0.59
T0 vs. 2 weeks	-2.2(-4.82 to 0.42)	0.11
T0 vs. 7 weeks	-0.1(-2.79 to 2.59)	>0.99
2 weeks vs. 7 weeks	2.1(-0.968 to 5.17)	0.21
**I**		
T0 vs. 96h	1.3(-1.79 to 4.39)	0.58
T0 vs. 2 weeks	-2.8(-8.24 to 2.64)	0.42
T0 vs. 7 weeks	5.1(0.344 to 9.86)	0.04
2 weeks vs. 7 weeks	7.9(3.78 to 12.0)	<0.001
**T**		
T0 vs. 96h	3.4(-0.270 to 7.07)	0.07
T0 vs. 2 weeks	0.2(-3.85 to 4.25)	>0.99
T0 vs. 7 weeks	6.9(3.31 to 10.5)	<0.001
2 weeks vs. 7 weeks	6.7(1.95 to 11.4)	0.008
**DC**		
T0 vs. 96h	-359(-366 to -351)	<0.001
T0 vs. 2 weeks	-363(-371 to -355)	<0.001
T0 vs. 7 weeks	-365(-371 to -358)	<0.001
2 weeks vs. 7 weeks	-1.4(-4.63 to 1.83)	0.56
**DQ**		
T0 vs. 96h	-346(-354 to -338)	<0.001
T0 vs. 2 weeks	-351(-359 to -342)	<0.001
T0 vs. 7 weeks	-151(-160 to -143)	<0.001
2 weeks vs. 7 weeks	200(199 to 201)	<0.001
**DP**		
T0 vs. 96h	-347(-355 to -338)	<0.001
T0 vs. 2 weeks	-350(-357 to -343)	<0.001
T0 vs. 7 weeks	-243(-248 to -238)	<0.001
2 weeks vs. 7 weeks	107(98.9 to 115)	<0.001
**DI**		
T0 vs. 96h	-344(-352 to -336)	<0.001
T0 vs. 2 weeks	-348(-354 to -343)	<0.001
T0 vs. 7 weeks	-29.8(-33.5 to -26.1)	<0.001
2 weeks vs. 7 weeks	319(314 to 323)	<0.001
**DT**		
T0 vs. 96h	-351(-357 to -345)	<0.001
T0 vs. 2 weeks	-356(-363 to -348)	<0.001
T0 vs. 7 weeks	-216(-224 to -208)	<0.001
2 weeks vs. 7 weeks	140(137 to 142)	<0.001

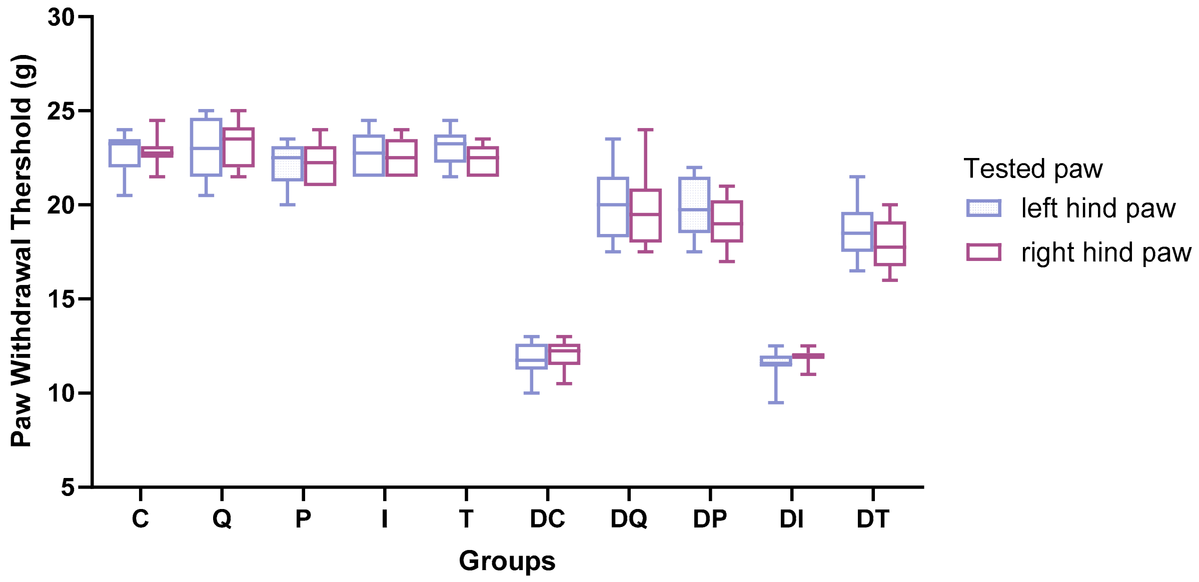

Supplementary Figure S1. Detailed presentation of measurements of left and right hind paws showing similar results between the left and right sides. n=10 animals per each group. Error bars indicate SD. SD: standard deviation.

Supplementary Table S7. Comparisons between groups with respect to their results in the paw withdrawal test (PWT) for mechanical nociceptive threshold determination. Measurements were done at week 7 after STZ/vehicle administration.

Compared groups	Mean Diff.(95.00% CI)	adj p-value
C vs. Q	-0.4(-1.65;0.853)	>0.99
C vs. P	0.62(-0.628;1.88)	0.85
C vs. I	0.09(-1.18;1.36)	>0.99
C vs. T	0(-1.25;1.25)	>0.99
C vs. DC	10.9(9.60;12.1)	<0.001
C vs. DQ	2.82(1.57;4.07)	<0.001
C vs. DP	3.23(1.97;4.48)	<0.001
C vs. DI	11(9.78;12.3)	<0.001
C vs. DT	4.5(3.25;5.75)	<0.001
Q vs. P	1.03(-0.228;2.28)	0.22
Q vs. I	0.49(-0.779;1.76)	0.97
Q vs. T	0.4(-0.853;1.65)	>0.99
Q vs. DC	11.3(10.0;12.5)	<0.001
Q vs. DQ	3.22(1.97;4.47)	<0.001
Q vs. DP	3.63(2.37;4.88)	<0.001
Q vs. DI	11.4(10.2;12.7)	<0.001
Q vs. DT	4.9(3.65;6.15)	<0.001
P vs. I	-0.53(-1.80;0.74)	0.94

P vs. T	-0.62(-1.88;0.63)	0.85
P vs. DC	10.2(8.97;11.5)	<0.001
P vs. DQ	2.2(0.942;3.45)	<0.001
P vs. DP	2.6(1.35;3.85)	<0.001
P vs. DI	10.4(9.16;11.7)	<0.001
P vs. DT	3.88(2.62;5.13)	<0.001
I vs. T	-0.09(-1.36;1.18)	>0.99
I vs. DC	10.8(9.49;12.0)	<0.001
I vs. DQ	2.73(1.46;4.00)	<0.001
I vs. DP	3.13(1.86;4.40)	<0.001
I vs. DI	10.9(9.67;12.2)	<0.001
I vs. DT	4.41(3.14;5.68)	<0.001
T vs. DC	10.9(9.60;12.1)	<0.001
T vs. DQ	2.82(1.57;4.07)	<0.001
T vs. DP	3.23(1.97;4.48)	<0.001
T vs. DI	11(9.78;12.3)	<0.001
T vs. DT	4.5(3.25;5.75)	<0.001
DC vs. DQ	-8.03(-9.28;-6.78)	<0.001
DC vs. DP	-7.63(-8.88;-6.37)	<0.001
DC vs. DI	0.19(-1.07;1.44)	>0.99
DC vs. DT	-6.35(-7.60;-5.10)	<0.001
DQ vs. DP	0.41(-0.85;1.66)	0.99
DQ vs. DI	8.22(6.96;9.47)	<0.001
DQ vs. DT	1.68(0.427;2.93)	0.001
DP vs. DI	7.81(6.56;9.06)	<0.001
DP vs. DT	1.28(0.02;2.53)	0.04
DI vs. DT	-6.54(-7.79;-5.28)	<0.001

Supplementary Table S8. Comparisons between groups with respect to SNCV and MNCV measurements. Measurements were done at week 7 after STZ/vehicle administration

	SNCV		MNCV	
**Compared groups**	**Mean Diff. (95.00% CI)**	**adj p-value**	**Mean Diff. (95.00% CI)**	**adj p-value**
C vs. Q	-0.4(-1.65;0.853)	>0.99	0.4(-1.49;2.20)	>0.99
C vs. P	0.63(-0.63;1.88)	0.85	0.8(-1.06;2.63)	0.93
C vs. I	0.09(-1.18;1.36)	>0.99	1.3(-0.57;3.12)	0.45
C vs. T	0(-1.25;1.25)	>0.99	0.9(-0.94;2.76)	0.85
C vs. DC	10.9(9.60;12.1)	<0.001	18.2(16.3;20.0)	<0.001
C vs. DQ	2.82(1.57;4.07)	<0.001	5.9(4.03;7.73)	<0.001

C vs. DP	3.23(1.97;4.48)	<0.001	8.3(6.45;10.1)	<0.001
C vs. DI	11(9.78;12.3)	<0.001	12.4(10.6;14.3)	<0.001
C vs. DT	4.5(3.25;5.75)	<0.001	9.5(7.68;11.4)	<0.001
Q vs. P	1.03(-0.23;2.28)	0.22	0.4(-1.42;2.28)	>0.99
Q vs. I	0.491(-0.78;1.76)	0.97	0.9(-0.94;2.76)	0.84
Q vs. T	0.4(-0.853;1.65)	>0.99	0.6(-1.30;2.40)	>0.99
Q vs. DC	11.3(10.0;12.5)	<0.001	17.8(16.0;19.7)	<0.001
Q vs. DQ	3.22(1.97;4.47)	<0.001	5.5(3.68;7.37)	<0.001
Q vs. DP	3.63(2.37;4.88)	<0.001	7.9(6.09;9.79)	<0.001
Q vs. DI	11.4(10.2;12.7)	<0.001	12.1(10.2;13.9)	<0.001
Q vs. DT	4.9(3.65;6.15)	<0.001	9.2(7.32;11.0)	<0.001
P vs. I	-0.53(-1.80;0.74)	0.94	0.5(-1.36;2.33)	>0.99
P vs. T	-0.62(-1.88;0.63)	0.85	0.1(-1.72;1.97)	>0.99
P vs. DC	10.2(8.97;11.5)	<0.001	17.4(15.5;19.2)	<0.001
P vs. DQ	2.2(0.942;3.45)	<0.001	5.1(3.25;6.94)	<0.001
P vs. DP	2.6(1.35;3.85)	<0.001	7.5(5.66;9.36)	<0.001
P vs. DI	10.4(9.16;11.7)	<0.001	11.6(9.78;13.5)	<0.001
P vs. DT	3.88(2.62;5.13)	<0.001	8.7(6.90;10.6)	<0.001
I vs. T	-0.09(-1.36;1.18)	>0.99	-0.4(-2.21;1.49)	>0.99
I vs. DC	10.8(9.49;12.0)	<0.001	16.9(15.1;18.8)	<0.001
I vs. DQ	2.73(1.46;4.00)	<0.001	4.6(2.77;6.46)	<0.001
I vs. DP	3.13(1.86;4.40)	<0.001	7(5.18;8.87)	<0.001
I vs. DI	10.9(9.67;12.2)	<0.001	11.1(9.29;13.0)	<0.001
I vs. DT	4.41(3.14;5.68)	<0.001	8.3(6.41;10.1)	<0.001
T vs. DC	10.9(9.60;12.1)	<0.001	17.3(15.4;19.1)	<0.001
T vs. DQ	2.82(1.57;4.07)	<0.001	5(3.13;6.82)	<0.001
T vs. DP	3.23(1.97;4.48)	<0.001	7.4(5.54;9.23)	<0.001
T vs. DI	11(9.78;12.3)	<0.001	11.5(9.65;13.3)	<0.001
T vs. DT	4.5(3.25;5.75)	<0.001	8.6(6.77;10.5)	<0.001
DC vs. DQ	-8.03(-9.28;-6.78)	<0.001	-12.3(-14.1;-10.4)	<0.001
DC vs. DP	-7.63(-8.88;-6.37)	<0.001	-9.9(-11.7;-8.03)	<0.001
DC vs. DI	0.185(-1.07;1.44)	>0.99	-5.8(-7.61;-3.92)	<0.001
DC vs. DT	-6.35(-7.60;-5.10)	<0.001	-8.7(-10.5;-6.80)	<0.001
DQ vs. DP	0.41(-0.85;1.66)	0.99	2.4(0.566;4.26)	0.002
DQ vs. DI	8.22(6.96;9.47)	<0.001	6.5(4.68;8.38)	<0.001
DQ vs. DT	1.68(0.427;2.93)	0.001	3.7(1.80;5.49)	<0.001
DP vs. DI	7.81(6.56;9.06)	<0.001	4.1(2.27;5.96)	<0.001
DP vs. DT	1.28(0.0220;2.53)	0.04	1.2(-0.614;3.08)	0.49
DI vs. DT	-6.54(-7.79;-5.28)	<0.001	-2.9(-4.73;-1.03)	<0.001

Supplementary Table S9. Comparisons between groups with respect to CMAP measurements. Measurements were done at week 7 after STZ/vehicle administration

Compared groups	Mean Diff. (95.00% CI)	adj p-value
C vs. Q	0.01(-0.522 to 0.542)	>0.99
C vs. P	0.198(-0.334 to 0.730)	0.97
C vs. I	-0.053(-0.585 to 0.479)	>0.99
C vs. T	-0.085(-0.617 to 0.447)	>0.99
C vs. DC	5.37(4.84 to 5.90)	<0.001
C vs. DQ	1.27(0.739 to 1.80)	<0.001
C vs. DP	1.97(1.43 to 2.50)	<0.001
C vs. DI	2.67(2.14 to 3.20)	<0.001
C vs. DT	1.74(1.21 to 2.27)	<0.001
Q vs. P	0.188(-0.344 to 0.720)	0.98
Q vs. I	-0.063(-0.595 to 0.469)	>0.99
Q vs. T	-0.095(-0.627 to 0.437)	>0.99
Q vs. DC	5.36(4.83 to 5.89)	<0.001
Q vs. DQ	1.26(0.729 to 1.79)	<0.001
Q vs. DP	1.96(1.42 to 2.49)	<0.001
Q vs. DI	2.66(2.13 to 3.19)	<0.001
Q vs. DT	1.73(1.20 to 2.26)	<0.001
P vs. I	-0.251(-0.783 to 0.281)	0.88
P vs. T	-0.283(-0.815 to 0.249)	0.78
P vs. DC	5.17(4.64 to 5.70)	<0.001
P vs. DQ	1.07(0.541 to 1.60)	<0.001
P vs. DP	1.77(1.24 to 2.30)	<0.001
P vs. DI	2.47(1.94 to 3.01)	<0.001
P vs. DT	1.54(1.01 to 2.07)	<0.001
I vs. T	-0.032(-0.564 to 0.500)	>0.99
I vs. DC	5.42(4.89 to 5.95)	<0.001
I vs. DQ	1.32(0.792 to 1.86)	<0.001
I vs. DP	2.02(1.49 to 2.55)	<0.001
I vs. DI	2.73(2.19 to 3.26)	<0.001
I vs. DT	1.79(1.26 to 2.32)	<0.001
T vs. DC	5.46(4.92 to 5.99)	<0.001
T vs. DQ	1.36(0.824 to 1.89)	<0.001
T vs. DP	2.05(1.52 to 2.58)	<0.001
T vs. DI	2.76(2.23 to 3.29)	<0.001
T vs. DT	1.82(1.29 to 2.35)	<0.001
DC vs. DQ	-4.1(-4.63 to -3.57)	<0.001
DC vs. DP	-3.4(-3.94 to -2.87)	<0.001
DC vs. DI	-2.7(-3.23 to -2.17)	<0.001
DC vs. DT	-3.63(-4.16 to -3.10)	<0.001
DQ vs. DP	0.695(0.163 to 1.23)	0.002
DQ vs. DI	1.4(0.869 to 1.93)	<0.001
DQ vs. DT	0.467(-0.0647 to 0.999)	0.14
DP vs. DI	0.706(0.174 to 1.24)	0.002
DP vs. DT	-0.228(-0.760 to 0.304)	0.93
DI vs. DT	-0.934(-1.47 to -0.402)	<0.001

Supplementary References

1. Chiș, I.C.; Clichici, A.; Simedrea, R.; Moldovan, R.; Lazar, V. (LEORDEAN); Clichici, S.; Oniga, O.; Nastasă, C. The Effects of a New Chromenyl-Methylene-Thiazolidine-2,4-Dione in Alleviating Oxidative Stress in a Rat Model of Streptozotocin-Induced Diabetes. *Stud. Univ. Babes-Bolyai Chem.* **2018**, *63*, doi:10.24193/subbchem.2018.4.08.
2. Canta, A.; Carozzi, V.A.; Chiorazzi, A.; Meregalli, C.; Oggioni, N.; Rodriguez-Menendez, V.; Sala, B.; Melcangi, R.C.; Giatti, S.; Lombardi, R.; et al. Multimodal Comparison of Diabetic Neuropathy in Aged Streptozotocin-Treated Sprague–Dawley and Zucker Diabetic Fatty Rats. *Biomedicines* **2023**, *11*, 20, doi:10.3390/biomedicines11010020.
3. Randall, L.O.; Selitto, J.J. A Method for Measurement of Analgesic Activity on Inflamed Tissue. *Arch. Int. Pharmacodyn. Ther.* **1957**, *111*, 409–419.
4. Zangiabadi, N.; Asadi-Shekaari, M.; Sheibani, V.; Jafari, M.; Shabani, M.; Asadi, A.R.; Tajadini, H.; Jarahi, M. Date Fruit Extract Is a Neuroprotective Agent in Diabetic Peripheral Neuropathy in Streptozotocin-Induced Diabetic Rats: A Multimodal Analysis. *Oxid. Med. Cell. Longev.* **2011**, *2011*, 976948, doi:10.1155/2011/976948.
5. Zangiabadi, N.; Mohtashami, H.; Hojatipour, M.; Jafari, M.; Asadi-Shekaari, M.; Shabani, M. The Effect of Angipars on Diabetic Neuropathy in STZ-Induced Diabetic Male Rats: A Study on Behavioral, Electrophysiological, Sciatic Histological and Ultrastructural Indices. *Sci. World J.* **2014**, *2014*, 721547, doi:10.1155/2014/721547.
6 Xu, X.; Zhang, D.; Liao, J.; Xiao, L.; Wang, Q.; Qiu, W. Galanin and Its Receptor System Promote the Repair of Injured Sciatic Nerves in Diabetic Rats. *Neural Regen. Res.* **2016**, *11*, 1517, doi:10.4103/1673-5374.191228.
7. Ota, A.; Kakehashi, A.; Toyoda, F.; Kinoshita, N.; Shinmura, M.; Takano, H.; Obata, H.; Matsumoto, T.; Tsuji, J.; Dobashi, Y.; et al. Effects of Long-Term Treatment with Ranirestat, a Potent Aldose Reductase Inhibitor, on Diabetic Cataract and Neuropathy in Spontaneously Diabetic Torii Rats. *J. Diabetes Res.* **2013**, *2013*, 175901, doi:10.1155/2013/175901.
8. Kambiz, S.; Neck, J.W. van; Cosgun, S.G.; Velzen, M.H.N. van; Janssen, J.A.M.J.L.; Avazverdi, N.; Hovius, S.E.R.; Walbeehm, E.T. An Early Diagnostic Tool for Diabetic Peripheral Neuropathy in Rats. *PLOS ONE* **2015**, *10*, e0126892, doi:10.1371/journal.pone.0126892.
